# Genetic polymorphisms contributing to hearing loss in children treated with platinum agents: a systematic review and meta-analysis protocol

**DOI:** 10.1136/bmjopen-2025-103735

**Published:** 2025-09-16

**Authors:** Lara Chavaz, Jakica Ćavar Pavić, Isabelle Dupanloup, Brice Fresneau, Hélène Cao Van, Nicolas Waespe, Yvonne Gloor, Marc Ansari

**Affiliations:** 1 CANSEARCH Research Platform for Paediatric Oncology and Haematology, Department of Paediatrics, Gynecology and Obstetrics, University of Geneva Faculty of Medicine, Geneva, GE, Switzerland; 2 Division of Paediatric Oncology and Haematology, University Hospitals of Geneva Department of Women-Children-Teenagers, Geneva, GE, Switzerland; 3 Division of Paediatric Oncology and Haematology, Department of Paediatrics, Inselspital University Hospital Bern Children’s Clinic, Bern, BE, Switzerland; 4 Graduate School for Cellular and Biomedical Sciences, University of Bern, Bern, BE, Switzerland; 5 Department of Children and Adolescent Oncology, Gustave Roussy Institute, Villejuif, Île-de-France, France; 6 U1018 CESP, Radiation Epidemiology team, Paris-Saclay University,Gustave Roussy Cancer Campus, INSERM, Villejuif, Île-de-France, France; 7 Division of Paediatric Otolaryngology Unit, Department of Otorhinolaryngology, Head and Neck Surgery, Geneva University Hospitals, Geneva, GE, Switzerland; 8 Audiology Laboratory, Department of Otorhinolaryngology, Head and Neck Surgery, Geneva University Hospitals, Geneva, GE, Switzerland; 9 Childhood Cancer Research Group, Institute of Social and Preventive Medicine & Department of Biomedical Research, University of Bern, Bern, GE, Switzerland

**Keywords:** PAEDIATRICS, Paediatric oncology, Hearing, CHEMOTHERAPY, GENETICS, Ototoxicity

## Abstract

**Abstract:**

**Introduction:**

The improved survival rates of children with cancer have heightened concerns about treatment-related chronic health conditions, including platinum-induced hearing loss (PIHL). Cisplatin and carboplatin, widely used in paediatric cancer therapies, frequently cause irreversible sensorineural hearing loss. PIHL affects 1.7–90.1% of patients exposed to these drugs, yet known risk factors—including age, cisplatin dosage, cranial radiation and co-treatment with ototoxic drugs—fail to fully explain interindividual variability. Genetic factors likely play a role in susceptibility to PIHL. Since genetic susceptibility in children may differ from adults, and given the critical window of auditory development, a focused investigation of paediatric genetic factors using quantitative methods is warranted to detect small to moderate effects and understand the polygenic nature of PIHL.

**Methods and analysis:**

In this study, we will systematically review and conduct a meta-analysis of genetic polymorphisms associated with PIHL in individuals diagnosed before the age of 21 years. Following Preferred Reporting Items for Systematic Reviews and Meta-analyses guidelines, the review will include randomised controlled trials, cohort, case-control and cross-sectional studies that analyse the genetic influence on PIHL in paediatric populations treated with cisplatin and carboplatin. A comprehensive search of PubMed, EMBASE and Cochrane databases will be conducted, supplemented by backward citation searching. Data will be extracted on study design, treatment details, hearing loss assessment methods, genetic findings and covariates. We will use forest plots to present the results, and both Mantel-Haenszel fixed-effects model and random-effects model will be used for meta-analysis. Heterogeneity will be assessed with the I² index. The study will address potential heterogeneity, individual study quality, proportion of missing data and meta-analysis bias. The quality of the evidence of the meta-analysis will be assessed using the Grading quality of evidence and strength of Recommendations (GRADE) approach.

**Discussion:**

This systematic review will enhance our understanding of the genetic contribution to PIHL in children and serve as a basis for further research for improvement of personalised treatment strategies for paediatric cancer care.

**PROSPERO registration number:**

CRD42024532664.

**Ethics and dissemination:**

All the included patient’s data are already published with an ethics approval for each study, respectively. No original data will be collected.

**STRENGTHS AND LIMITATIONS OF THIS STUDY**

- The strength of this systematic review and meta-analysis is its exclusive focus on the paediatric population and its quantitative approach, which may help uncover small to moderate genetic effects contributing to the polygenic nature of platinum-induced hearing loss in a patient population with specific characteristics.
- Variability in ototoxicity grading scales limits comparability across studies; to address this, we will apply a dichotomous classification (hearing loss: yes/no) and perform sensitivity analyses by severity and grading system where feasible.
- All clinical covariates related to platinum-induced hearing loss risk will be narratively summarised, with sensitivity analyses conducted to assess the robustness of results.

## Introduction

 The overall survival of children suffering from cancer has significantly improved in recent years, leading to increased awareness of chronic health conditions (CHCs) in childhood cancer survivors (CCS). Among CHCs, ototoxicity secondary to platinum-containing chemotherapy exposure, referred to as platinum-induced hearing loss (PIHL), tinnitus or vertigo, is a well-recognised complication in CCS.^1^ Cisplatin and carboplatin are frequently used to treat hepatoblastoma, neuroblastoma, osteosarcoma and brain tumours in children. These drugs impair inner ear function by targeting three major structures in the cochlea: hair cells, connective tissues (stria vascularis and spiral ligament) and spiral ganglion neurons, resulting in irreversible, bilateral sensorineural hearing loss.^2 3^ The unique characteristics of cochlear tissue, specifically its inability to regenerate and the prolonged retention of cisplatin for several years, complicate our understanding of the mechanisms leading to PIHL.^4^

Several key enzymes have been implicated as mediators of PIHL. ^5 6^The *ACYP2* gene, expressed in the cochlea, is involved in maintaining calcium homeostasis, a crucial signalling pathway for hair cell development and apoptosis.^7^,^9^ thiopurine S-methyltransferase (TPMT) and Catechol-O-methyltransferase (COMT) encode methyltransferases that rely on S-adenosylmethionine (SAM) as a substrate, and elevated SAM levels have been shown to amplify cisplatin-induced toxicity.^10^,^12^

From a clinical perspective, recent cohort studies of CCS indicate that PIHL rates range between 1.7% and 90.1% in exposed patients.^13^ Children are more vulnerable than adults to PIHL due to the ongoing maturation of their auditory pathways.^14^ This ontogenetic susceptibility, combined with the detrimental consequences of hearing loss on child language development, speech acquisition and social integration, emphasises the importance of paediatric-specific approaches to ototoxicity management.^15 16^ Known clinical risk factors for PIHL are the use of cisplatin compared with carboplatin, higher cisplatin dose intensity, younger age at exposure, concomitant cranial radiation, renal insufficiency and administration of other ototoxic drugs (eg, aminoglycoside antibiotics, furosemide loop diuretics and vincristine).^1017^,^19^

However, these risk factors only partly explain the heterogeneity in the onset and severity of PIHL between patients. Genetic predispositions likely contribute to this variability, and the inconsistent findings across studies may reflect modest individual effects within a broader polygenic framework.^20^,^22^ Previous systematic reviews have combined adult and paediatric populations,^23^,^26^ with only one 2017 meta-analysis focusing exclusively on children, limited to *TPMT*, *COMT* and *ACYP2* genes.^27^ The most recent systematic review was published in 2023^23^ and offered key insights into genetic susceptibility to PIHL across age groups. While only a few additional paediatric studies have emerged,^10 28 29^ this review builds on previous work by focusing specifically on children and taking a quantitative approach to detect small to moderate effects and clarify its polygenic nature.

## Aims and objectives

The aim of this paper is to describe the protocol for conducting a systematic review and meta-analysis of the literature in which genetic polymorphisms have been tested for an association with PIHL in children. To this end, the proposed systematic review will focus on:

- Collecting and analysing genetic variants tested for an association with PIHL in children, including: (1) all variants reported as significantly associated in at least one candidate gene study (p<0.05) or genome-wide association studies (GWAS) and large-scale candidate gene study (using author-defined correction or Bonferroni correction or p<5×10⁻⁸ if not specified) and (2) the corresponding association results—regardless of significance—from all other independent study populations analysing the same variant.
- Performing a meta-analysis of all variants reported as significant in more than one independent paediatric study population, to enable detection of small to moderate effects and clarify the polygenic nature of PIHL.
- Reporting on non-genetic covariates investigated in relation to PIHL in children.

## Methods

This systematic review and meta-analysis will be conducted and reported following the Preferred Reporting Items for Systematic Reviews and Meta-analyses (PRISMA) guidelines.^30^

### Prospero registration

We searched the International Prospective Register of Systematic Reviews (PROSPERO) register for ongoing systematic reviews dealing with the same topic in June 2024. No similar review was identified. Thus, in accordance with the guidelines, we registered our systematic review protocol on PROSPERO on 14 July 2024 (registration number CRD42024532664).

### Eligibility criteria

The table 1 outlines the eligibility criteria described in detail below.

**Table 1:** Study inclusion and exclusion criteria

		Inclusion criteria	Exclusion criteria
Study characteristics	Study design	Randomised controlled trials	Preprints article Conference abstracts
		Observational studies (cohort studies, cross-sectional studies)	
		Case-control studies; case-series >20 patients	Book chapters
			Case reports or case-series <20 patients
			Reviews
	Languages	English	Others
		German	
		French	
		Italian	
		Spanish	
		Swedish	
		Danish	
		Norwegian Croatian	
Report characteristics	Participants	Human population Paediatric population, half of the cohort 21 years old at diagnosis	Animals or cells Majority of adult population
	Exposition	Exposure to carboplatin or cisplatin-based chemotherapy	No carboplatin or cisplatin exposure
	Comparators	Genetic polymorphisms	No genetic analysis
	Outcomes	Objectively measured hearing loss	No documentation of hearing loss

#### Study design, languages and date of publication

We will include randomised controlled trials and observational studies such as cohort studies (prospective and retrospective), cross-sectional studies and case-control studies, as well as case-series with more than 20 patients. We will exclude preprints, conference abstracts, books or book chapters, case reports or case series including less than 20 patients and reviews. Studies published in English, German, French, Italian, Spanish, Swedish, Croatian, Danish and Norwegian—the languages spoken by our research team—will be included. No restriction by date of publication will be applied.

#### Participants

We will only include studies conducted on human participants, focusing predominantly on paediatric population. For articles that include both paediatric and adult patients, at least half of the cohort must have been diagnosed at age 21 years or younger to be eligible for inclusion, unless data from paediatric patients are reported separately.

#### Exposure

Participants must have been exposed to cisplatin and/or carboplatin. Cisplatin and carboplatin exposure will be considered, regardless of the cumulative dose received, the timing between doses and the treatment duration.

#### Comparison

We will include studies reporting genetic results of precisely defined polymorphisms from candidate gene or GWAS.

We will include all variants significantly associated with PIHL in at least one candidate gene study or GWAS, using an adjusted p value<0.05. Significance will be defined using author-defined multiple testing correction. If no multiple test correction is provided, we will apply Bonferroni correction or the conventional genome-wide threshold (p<5×10⁻⁸) for GWAS. For each included variant, we will extract association results—regardless of significance—from all other independent study populations where the same variant was analysed. If a large-scale study (>100 gene variants tested) does not report genome-wide significant findings, we will consider using variants meeting the suggestive threshold (p<1×10⁻⁵).

#### Outcomes

The primary outcome is the association between genetic polymorphisms and PIHL in children, measured by pure-tone or play audiometry. PIHL is defined as bilateral, often permanent sensorineural hearing loss, primarily affecting high frequencies and worsening with platinum dose intensity.^14 31 32^ Various ototoxicity grading systems are used in children such as ASHA,^33^ CTCAE (V.5.0 or earlier),^34^,^38^ Brock, Chang, Muenster or SIOP Boston scales (table 2).^33 39 40^ Moreover, various classification schemes are used across studies to distinguish between hearing loss and no hearing loss (eg, G0 vs G≥1; G0–1 vs G≥2; G0 vs G≥2 excluding G1), with grade 1 being the most heterogeneously classified. In this study, the outcome hearing loss will be dichotomised as present or absent (yes/no), as reported in each independent study population. We will include studies reporting hearing loss based on recognised ototoxicity grading scales (table 2) or based on the reported use of hearing aids after treatment when no formal grading is available.

**Table 2:** Ototoxicity classification systems in children with cut-off points of each of the scale for the diagnosis of hearing loss

Scale denomination	Method	Version of the scale	Normal hearing	Any hearing loss
ASHA^33^	Based on threshold changes from baseline	–	No hearing change from baseline	>10 dB change from baseline at two consecutive frequencies or ≥20 dB change at 1-frequency, or loss of response where one was previously obtained.
Common Terminology Criteria for Adverse Events	Based on threshold changes from baseline	V.5.0^34^	Controls: threshold shift ≤20 dB HL at all frequencies	Grade 1: threshold shift >20 dB HL (ie, 25 dB HL or greater) at >4 kHz in at least one ear Grade 2: threshold shift >20 dB at 4 kHz in at least one ear Grade 3: HL sufficient to indicate therapeutic intervention, including hearing aids; threshold shift >20 dB at two to <4 kHz in at least one ear Grade 4: audiological indication for cochlear implant; >40 dB HL (ie, 45 dB HL or more) at ≥2 kHz
		V,4.03^35^; V.4.0^36^	Controls: threshold shift ≤20 dB HL at all frequencies	Grade 1: threshold shift >20 dB HL at 8 kHz (or at any frequency for v4.0) in at least one ear Grade 2: threshold shift >20 dB at ≥4 kHz (or at >4 kHz for V.4.0) in at least one ear Grade 3: HL sufficient to indicate therapeutic intervention, including hearing aids; threshold shift >20 dB at ≥3 kHz in at least one ear; additional speech-language related services indicated Grade 4: audiological indication for cochlear implant and additional speech-language related services indicated
		V.3.0^37^	Controls: threshold shift <15 dB HL at all frequencies	Grade 1: threshold shift or loss of 15–25 dB relative to baseline, averaged at two or more contiguous test frequencies in at least one ear or subjective change in the absence of a grade 1 threshold shift Grade 2: threshold shift or loss of >25–90 dB, averaged at two contiguous test frequencies in at least one ear Grade 3: HL sufficient to indicate therapeutic intervention, including hearing aids Grade 4: audiological indication for cochlear implant and requiring additional speech-language related services
		V.2.0^38^	Controls: no HL	Grade 1: HL on audiometry only Grade 2: tinnitus or HL, not requiring hearing aid or treatment Grade 3: tinnitus or HL, correctable with hearing aid or treatment Grade 4: several unilateral or bilateral HL (deafness), not correctable
Brock^33 40^	Based on absolute hearing level	–	Grade 0: <40 dB HL at all test frequencies	Grade 1: ≥40 dB HL at 8 kHz only Grade 2: ≥40 dB HL at≥4 kHz Grade 3: ≥40 dB HL at≥2 kHz Grade 4: ≥40 dB HL at≥1 kHz
Chang^40^	Based on absolute hearing level	–	Grade 0: ≤20 dB HL at 1 kHz, 2 kHz and 4 kHz	Grade 1 a: ≥40 dB HL at 6–12 kHz Grade 1b: >20 dB and <40 dB HL at 4 kHz Grade 2 a: ≥40 dB HL at ≥4 kHz; Grade 2b: >20 dB and <40 dB HL at 1 kHz, 2 kHz or 3 kHz Grade 3: ≥40 dB HL at ≥2kHz or 3 kHz Grade 4: ≥40 dB HL at ≥1 kHz
Boston SIOP^32^,^33 40^	Based on absolute hearing level	–	Grade 0: ≤20 dB HL at all frequencies	Grade 1: >20 dB HL at >4 kHz Grade 2: >20 dB HL at ≥4 kHz Grade 3: >20 dB HL at ≥2 kHz or 3 kHz Grade 4: >40 dB HL at ≥2 kHz
Muenster^40^	Based on absolute hearing level		Grade 0: ≤10 dB HL at all frequencies	Grade 1: >10 dB at ≤2 kHz Grade 2a: >20 ≤40 dB at ≥4 kHz; Grade 2b: >40 ≤60 dB at ≥4 kHz; Grade 2c: >60 dB at ≥4 kHz Grade 3a: >20 ≤40 dB at <4 kHz; Grade 3b: >40 ≤60 dB at <4 kHz; Grade 3c: >60 <80 dB at <4 kHz Grade 4: ≥80 dB at <4 kHz

ASHA, American Speech-Language-Hearing Association ; dB, decibels; HL, hearing loss; kHz, kilo Hertz; SIOP, Society of Pediatric Oncology.

The secondary outcome includes the association of non-genetic covariates with PIHL, such as age at treatment, details of the platinum regimen, cranial radiation exposure and exposure to other ototoxic drugs. For each covariate, we will report whether statistical testing was performed and whether a statistically significant association was observed.

### Search strategy

A systematic search will be conducted on PubMed, EMBASE and the Cochrane Central Register of Controlled Trials databases at the start of the systematic review with at least one update prior to manuscript submission. We have decided not to supplement the electronic databases search with results coming from the grey literature.

The search strategy was developed by the project team and reviewed by an independent Health Sciences Librarian following Peer Review of Electronic Search Strategies guidelines.^41^ It used medical subject headings, text words and synonyms related to PIHL in paediatric patients and genetic polymorphisms, with language filters applied. Based on Population, Intervention, Comparison, Outcome (PICO) criteria, the strategy was refined using PubMed and adapted for other databases (online supplemental table 1) with the SR-accelerator software (https://sr-accelerator.com/#/methods-wizard). To validate its effectiveness, the strategy was tested against 19 relevant articles from a prior systematic review,^24^ all of which were successfully retrieved.

### Data collection and extraction

#### Screening

Two reviewers will independently screen the titles and abstracts of each manuscript for eligibility using Rayyan, a web-available software specifically designed to coordinate systematic literature reviews (https://www.rayyan.ai). Any disagreements will be resolved by an independent reviewer. The reasons for manuscript exclusion will be documented in all cases. After completion of the screening process, the reference list of each included study will be searched for eligible studies not identified by the systematic search. For any newly identified manuscripts, the same screening procedure will be applied to determine further eligible studies.

#### Data extraction

Full reports of all included manuscripts will be examined for data extraction by a first reviewer. A second reviewer will check the extracted data. Any disagreements will be resolved by a third reviewer with expertise in the relevant domain (eg, genetics, clinical oncology). All extracted data will be compiled in an Excel spreadsheet. We will collect data about the study design, number of study sites, participating countries, clinical trial registration number, inclusion and exclusion criteria, population description, clinical characteristics of the population (age at platinum exposure, gender, ethnicity, cancer types), exposure type (cisplatin, carboplatin exposure, treatment protocol), genotyping (sequencing method, list of selected genetic variants, number of variants included in final analysis and, in case of GWAS, genetic data quality control), outcome measurement (hearing loss by ototoxicity scales as reported, time since diagnosis, audiometric techniques, hearing status at baseline), tinnitus and vertigo symptoms, length of follow-up to outcome measurement, data metrics and statistical analysis (cohort size, effect and variability measures, statistical methods used, multiple test correction), covariates and their inclusion in multivariable models (age at time of platinum treatment, cumulative dose of platinum compound received (mg/m^2^), maximum dose of platinum compound (mg/m^2^) per day, number of cycles of platinum compound, dosing intervals, cranial radiation, concomitant vincristine chemotherapy, concomitant aminoglycoside antibiotics and/or furosemide loop diuretics treatments, renal insufficiency before platinum treatment and administration of prophylactic treatments such as amifostine, sodium thiosulfate and N-acetylcysteine).

#### Missing data

Missing data for each individual study, as well as the methods used to handle them, will be reported. If essential information is unavailable, corresponding authors will be contacted to request additional data.

#### Quality and validity assessment

Two reviewers will independently assess the quality of each included study using the Q-Genie tool, a validated instrument for genetic association studies.^42^ In the case of disagreement, a third reviewer will resolve the conflict. Genotyping quality metrics—such as sequencing method, call rates, genotyping procedure, Hardy-Weinberg Equilibrium and multiple test corrections (eg, Bonferroni, False Discovery Rate)—will be reported descriptively to complement the Q-Genie assessment and ensure alignment with Strengthening the Reporting of Genetic Association recommendations, on which Q-Genie is partly based.^43^ A global validity and quality assessment statement will be issued for each study.

### Data synthesis and re-analysis

#### Synthesis

Studies will be grouped and summarised based on their context, with demographic and genetic data presented in tabular form. Study quality and validity, genotyping quality and the proportion of missing data will be detailed in a supplementary table. For each genetic variant, the number of patients with and without hearing loss will be extracted—as reported in each independent study population—and we will include its reported univariate analysis results [OR, 95% CI, p value].

Non-genetic covariates will be reported narratively, including whether they were assessed statistically. Where available, we will report subgroup or stratified ORs as reported in the original studies, to explore potential effect modification by clinical covariates (eg, age at treatment, cranial radiation, platinum exposure, concomitant ototoxic drugs).

#### Re-analysis

For independent study populations in which the exact number of patients per ototoxicity grade is available, we will recategorise the data according to the different classification schemes used in the meta-analysis (eg, G0 vs G≥1, G0–1 vs G≥2, G0 vs G≥2 excluding G1) to maximise comparability. We will then reanalyse the data using χ^2^ or Fisher’s exact tests to calculate the OR, 95% CI and p value. Re-analysis will also be performed for studies that report only the number of patients per variant without providing the OR or that report a non-OR effect measure (eg, risk ratio, HR). All values derived from a recategorisation will be clearly indicated as such.

### Data analysis

#### Meta-analysis and assessment of heterogeneity

Each genetic variant that has been reported as significantly associated with PIHL in more than one independent study population will be considered for the meta-analysis. We will conduct a meta-analysis of the dichotomous outcome, hearing loss, using both the Mantel-Haenszel fixed-effects model and the random-effects model to account for between study variance τ^2^.^44^ Heterogeneity between studies will be assessed using the inconsistency index (I^2^), which measures the percentage of variability across all studies and the Cochran’s Q test.^44^ To investigate the sources of heterogeneity, we will perform sensitivity analyses based on common risk factors for PIHL. Results will be summarised using forest plots to facilitate comparisons across studies for the same variant. For each forest plot, the pooled OR for hearing loss associated with a genetic variant will be calculated. The precision of the pooled OR will be expressed as a 95% CI.

All analyses will be conducted using R software (V.2024.12.0+467 or later) with appropriate statistical packages.

#### Robustness assessment

Sensitivity analyses will be conducted whenever possible to assess the robustness of our findings. These will include stratifications by study quality, presence of non-genetic covariates (eg, age at treatment, cranial radiation, platinum exposure, concomitant ototoxic drugs) and phenotype definition. The latter will consider how ototoxicity grades are handled (eg, G0 vs G≥1, G0–1 vs G≥2, G0 vs G≥2 excluding G1 and no hearing aids vs use of hearing aids). If data allow, analyses stratified by ototoxicity grading system will be performed.

#### Meta-biases

We will assess potential outcome reporting bias. If a study protocol is available, we will compare the outcomes reported in the protocol to those reported in the published manuscript.

We recognise the inherent limitations in assessing publication bias. Negative genetic association studies are less likely to be published, which may introduce bias. These limitations will be considered when interpreting our results.

#### Confidence in cumulative evidence

The overall confidence in the evidence will be assessed using the GRADE tool, evaluating five domains: risk of bias, inconsistency, indirectness, imprecision and publication bias.^45^ Evidence quality will be categorised as high, moderate, low or very low. The ‘Summary of Findings’ tables will present each genetic variant, its pooled OR for hearing loss, the number of contributing studies and participants and the corresponding GRADE assessment

## Discussion

This protocol details the methodology of our systematic review and meta-analysis, aiming to summarise current knowledge on genetic variants influencing PIHL risk in children and fulfils all requirements of the^46^ PRISMA protocols 2015 checklist (online supplemental table 2).^46^ By focusing on the paediatric population and taking a quantitative approach, this review addresses the possibility of unique genetic ototoxicity risk variants in this group that may not have been detected in studies combining adult and paediatric populations. Additionally, the review may help detect small to moderate effects, clarify the polygenic nature of PIHL.

For this study, the primary outcome of interest is hearing loss, as a manifestation of ototoxicity. Other ototoxic symptoms such as tinnitus and vertigo are not routinely assessed in paediatric clinical practice and are therefore less frequently reported, despite the existence of vestibular function tests. Consequently, tinnitus and vertigo will not be considered as predefined outcomes in this analysis; however, if reported in the included studies, they will be documented in the data synthesis.

Concerning platinum agent exposure, oxaliplatin exposure was considered, but due to the absence of additional results when this term was added to our search equation, we removed it from the final search equation.

To evaluate reproducibility, we will identify variants initially reported as significant in at least one independent study population and examine their association results in other included datasets analysing the same variant, regardless of significance. This approach facilitates cross-validation of previously reported associations across diverse study populations, though we acknowledge a potential risk of selection bias.

This meta-analysis will use both fixed-effects (Mantel-Haenszel method) and random-effects models to account for study size and between-study variance. Given the polygenic nature of PIHL, all pooled ORs will be reported, including modest associations. Formal assessment of publication bias (eg, Funnel plot, Egger’s test) is unlikely due to the limited number of studies per variant, but potential bias—including underreporting of non-significant findings—will be considered. The Q-Genie tool^42^ will be applied to all included studies. For those previously assessed with Q-Genie,^23^ we will compare scores to ensure consistency and maintain methodological rigour across the evidence base.

A major limitation of this systematic review is the lack of consensus on validated paediatric ototoxicity scales, which assess either threshold changes from baseline or absolute hearing level. While baseline data are ideal, they are often missing, making absolute thresholds more commonly reported. Hearing data with baseline assessment will inform quality appraisal but not eligibility. To enable meta-analysis across studies using different grading systems, hearing loss will be dichotomised as present or absent, based on each study’s original classification. This inclusive strategy increases power but may introduce heterogeneity, particularly regarding the handling of grade 1, which has variable clinical relevance. Although overall concordance between grading systems is generally good,^40^ discrepancies persist, especially in early grades. To explore the impact of different phenotype definitions, a series of sensitivity analyses will be conducted as outlined in the methods. This approach aims to balance inclusivity with phenotypic clarity and may help inform future standards for case/control definitions in genetic studies of PIHL.

Another important limitation of our study is the way cisplatin exposure is commonly reported. Although dose intensity is more relevant for assessing concentration-dependent toxicities such as PIHL,^10^ most studies report only cumulative dose. Therefore, when dose intensity data are unavailable, we will extract cumulative dose, while acknowledging that it may not accurately capture the timing or extent of exposure most relevant to hearing loss onset and severity. This limitation, which is shared by many genetic association studies on PIHL, should be carefully considered when interpreting our results.

A key challenge in this meta-analysis is the potential overlap of patient populations across studies, as most genetic risk studies on PIHL in children are retrospective and based on limited national cohorts. To address this, detailed population data will be extracted, and in cases of ambiguity, corresponding authors will be contacted to clarify overlaps and avoid data duplication.

## Supplementary material

10.1136/bmjopen-2025-103735 online supplemental file 1

10.1136/bmjopen-2025-103735 online supplemental file 2
