# Supplementary material for: Genetic polymorphisms contributing to hearing loss in children treated with platinum agents: a systematic review and meta-analysis protocol
Source: BMJ Open. 2025 Sep 16;15(9):e103735. doi: 10.1136/bmjopen-2025-103735 (PMC12443194; doi:10.1136/bmjopen-2025-103735)
Supplement: online supplemental file 1 [file bmjopen-15-9-s001.docx]

| ***Database*** | ***Search equation*** |
| --- | --- |
| ***PubMed*** | *("Child"[Mesh] OR "Infant"[Mesh] OR "Adolescent"[Mesh] OR "Young adult"[Mesh] OR “Pediatrics”[Mesh] OR "AYA"[Title/Abstract] OR "young adult*" [Title/Abstract] OR "Child*" [Title/Abstract] OR "Infant*" [Title/Abstract] OR "Adolescent*" [Title/Abstract] OR “Pediatric*”[Title/Abstract] OR paediatric*[Title]) AND ("Cisplatin"[Mesh] OR "Carboplatin"[Mesh] OR "Platinum"[Mesh] OR “Drug Therapy”[Mesh] OR "Cisplatin"[Title/Abstract] OR "Carboplatin"[Title/Abstract] OR "Platinum"[Title/Abstract] OR "Drug Therapy"[Title/Abstract] OR "Cis-diamminedichloroplatinum*"[tiab] OR “Dichlorodiammineplatinum*”[tiab] OR “Biocisplatinum”[tiab] OR “Chemotherap*”[tiab] OR "CBDCA" [tiab]) AND ("Pharmacogenomic Variants"[Mesh] OR "Pharmacogenetics"[Mesh] OR "Genetic Predisposition to Disease"[Mesh] OR "Genes"[Mesh] OR "Genotype"[Mesh] OR "Polymorphism, Single Nucleotide"[Mesh] OR “Mutation”[Mesh] OR "Genetic Association Studies"[Mesh] OR "Genetic Markers"[Mesh] OR “Genetic variant*”[tiab] OR “SNPs”[tiab] OR “Genetic Susceptibilit*”[tiab] OR “Pharmacogenomic*”[tiab] OR "pharmacogenomic variant*"[tiab] OR "genetic polymorphism*" OR "pharmacogenetic*"[tiab] OR "Genetic Predisposition to Disease*"[tiab] OR "Genes"[tiab] OR "Gene"[tiab] OR "Genotyp*"[tiab] OR "Mutation*"[tiab] OR "Genetic Association Stud*"[tiab] OR "Genetic Marker*"[tiab] OR "Genetic vulnerabilit*" [tiab] OR "Polymorphism Single Nucleotide"[Title/Abstract:~1]) AND ("Ototoxicity"[Mesh] OR “Hearing Loss"[Mesh] OR “Audiometry”[Mesh] OR “Hearing”[Mesh] OR “Cochlea”[Mesh] OR "Drug-Induced Ototoxicity"[Title/abstract:~1] OR "Drug-Related Otological Toxicity"[Title/abstract:~1] OR "Drug-induced Cochleotoxicity"[Title/abstract:~1] OR "Cisplatin induced hearing loss"[Title/Abstract:~1] OR “Deafness”[tiab] OR “CIHL”[tiab] OR "Hypoacusi*"[tiab] OR "Hearing"[tiab] OR “audiometry”[tiab] OR “cochlea”[tiab])* |
| ***Embase*** | *('child'/exp OR 'infant'/exp OR 'adolescent'/exp OR 'young adult'/exp OR 'pediatrics'/exp OR aya:ti,ab,kw OR 'young adult*':ti,ab,kw OR child*:ti,ab,kw OR infant*:ti,ab,kw OR adolescent*:ti,ab,kw OR p$ediatric*:ti,ab,kw) AND ('cisplatin'/exp OR 'carboplatin'/exp OR 'platinum'/exp OR 'drug therapy'/exp OR cisplatin:ti,ab,kw OR carboplatin:ti,ab,kw OR platinum:ti,ab,kw OR 'drug therapy':ti,ab,kw OR 'cis diamminedichloroplatinum*':ti,ab,kw OR dichlorodiammineplatinum*:ti,ab,kw OR biocisplatinum:ti,ab,kw OR chemotherap*:ti,ab,kw OR cbdca:ti,ab,kw) AND ('pharmacogenomic variant'/exp OR 'pharmacogenetics'/exp OR 'genetic predisposition'/exp OR 'gene'/exp OR 'genotype'/exp OR 'single nucleotide polymorphism'/exp OR 'mutation'/exp OR 'genetic association study'/exp OR 'genetic marker'/exp OR 'genetic polymorphism'/exp OR 'genetic variant*':ti,ab,kw OR snps:ti,ab,kw OR 'genetic susceptibilit*':ti,ab,kw OR pharmacogenomic*:ti,ab,kw OR 'pharmacogenomic variant*':ti,ab,kw OR pharmacogenetic*:ti,ab,kw OR 'genetic predisposition to disease*':ti,ab,kw OR gene:ti,ab,kw OR genes:ti,ab,kw OR genotyp*:ti,ab,kw OR mutation*:ti,ab,kw OR 'genetic association stud*':ti,ab,kw OR 'genetic marker*':ti,ab,kw OR 'genetic vulnerabilit*':ti,ab,kw OR 'genetic polymorphism*':ti,ab,kw OR (polymorphism NEAR/2 single NEAR/2 nucleotide)) AND ('hearing'/exp OR 'hearing disorder'/exp OR 'ototoxicity'/exp OR 'audiometry'/exp OR 'cochlea'/exp OR (drug NEAR/2 induced NEAR/2 ototoxicity) OR (drug NEAR/2 related NEAR/2 otological NEAR/2 toxicity) OR (drug NEAR/2 induced NEAR/2 cochleotoxicity) OR (cisplatin NEAR/2 induced NEAR/2 hearing NEAR/2 loss) OR deafness:ti,ab,kw OR cihl:ti,ab,kw OR hypoacusi*:ti,ab,kw OR hearing:ti,ab,kw OR audiometry:ti,ab,kw OR cochlea:ti,ab,kw) NOT ('conference abstract'/it OR 'conference review'/it OR 'conference paper'/it) AND ([danish]/lim OR [english]/lim OR [french]/lim OR [german]/lim OR [icelandic]/lim OR [italian]/lim OR [norwegian]/lim OR [spanish]/lim OR [swedish]/lim) NOT ([animals]/lim NOT [humans]/lim)* |
| ***Cochrane*** | *(aya OR «young adult*» OR child* OR infant* OR adolescent* OR pediatric* OR paediatric*) AND (cisplatin OR carboplatin OR platinum OR «drug therapy» OR «cis diamminedichloroplatinum*» OR dichlorodiammineplatinum* OR biocisplatinum OR chemotherap* OR cbdca) AND («genetic variant*» OR snps OR «genetic susceptibilit*» OR pharmacogenomic* OR «pharmacogenomic variant*» OR pharmacogenetic* OR «genetic predisposition to disease*» OR gene OR genes OR genotyp* OR mutation* OR «genetic association stud*» OR «genetic marker*» OR «genetic vulnerabilit*» OR «genetic polymorphism*» OR (polymorphism NEAR/2 single NEAR/2 nucleotide)) AND ((drug NEAR/2 induced NEAR/2 ototoxicity) OR (drug NEAR/2 related NEAR/2 otological NEAR/2 toxicity) OR (drug NEAR/2 induced NEAR/2 cochleotoxicity) OR (cisplatin NEAR/2 induced NEAR/2 hearing NEAR/2 loss) OR deafness OR cihl OR hypoacusi* OR hearing OR audiometry OR cochlea)* |

***Supplemental Table 1* –** Literature database search equations.
