# Supplementary material for: Genetic polymorphisms contributing to hearing loss in children treated with platinum agents: a systematic review and meta-analysis protocol
Source: BMJ Open. 2025 Sep 16;15(9):e103735. doi: 10.1136/bmjopen-2025-103735 (PMC12443194; doi:10.1136/bmjopen-2025-103735)
Supplement: Supplementary file 3 [file bmjopen-2025-103735.reviewer_comments.pdf]

PEER REVIEW HISTORY

BMJ Open publishes all reviews undertaken for accepted manuscripts. Reviewers are asked to complete a checklist review form and are provided with free text boxes to elaborate on their assessment. These free text comments are reproduced below.

ARTICLE DETAILS

Title (Provisional)

Genetic polymorphisms contributing to hearing loss in children treated with platinum agents: a systematic review and meta-analysis protocol

Authors

Chavaz, Lara; Ćavar Pavić, Jakica; Dupanloup, Isabelle; Fresneau, Brice; Cao Van, Hélène; Waespe, Nicolas; Gloor, Yvonne; Ansari, Marc

---

VERSION 1 - REVIEW

**Reviewer**	**1**
**Name**	**Carleton, Bruce**
**Affiliation**	**University of British Columbia**
**Date**	**02-Jun-2025**
**COI**	**None**

Reviewer comments to authors:

This review was conducted by Drs. Bruce Carleton and Catrina Loucks.

In this work, the authors provide a detailed protocol that will specifically quantify the impact of genetic variants on the likelihood of developing platinum-induced ototoxicity in children. The authors should leverage from some of the recent work of previous systematic reviews. For example, a recent systematic review conducted by our group (Scott, E. N. *et al.* Systematic Critical Review of Genetic Factors Associated with Cisplatin-induced Ototoxicity: Canadian Pharmacogenomics Network for Drug Safety 2022 Update. *Ther Drug Monit* (2023) doi:10.1097/FTD.0000000000001113) was cited by the authors primarily to highlight that a similar review to that proposed has not been conducted (our review included both children and adults). However, our review would have included most, if not all, of the relevant pediatric studies (up to June 2020) with the caveat that we did not consider carboplatin-specific studies (but these studies are likely to be few if any since carboplatin is mostly used after cisplatin has already been provided). Comparing pediatric studies retrieved in our study to those retrieved by their search strategy will help determine what the new study will provide compared to the existing one. Furthermore, while study quality should be assessed, our prior study already assessed the quality of several studies likely to be included in their work using a tool designed for genetic association studies (Sohani, Z. N. *et al.* Assessing the quality of published genetic association studies in meta-analyses: the quality of genetic studies (Q-Genie) tool. *BMC Genet* **16**, 50 (2015)). Finally, it is likely that the combined impact for many variants will lead to a relatively modest effect size (especially given the inherent heterogeneity

between studies). Therefore, it is important to provide more rationale on the value of quantifying the magnitudes of effect (when many are likely to be small). For example, in our prior review, we chose to highlight only variants that had been associated with ototoxicity in at least three independent patient populations with magnitudes of effect (i.e., odds ratios) greater than 3 (or less than 0.33) due to the clinical importance of such strong effect sizes. Given that published manuscripts differ with respect to publishing requirements, it is essential not to compare "published studies" but populations of patients to determine if findings have been replicated. Nature Genetics for example requires two independent populations and biological validation. This is quite a bit more stringent that many other journals.

Overall, while the protocol for this proposed review is well-described, it is unclear whether this review will net much new information since this topic has already been reviewed recently (2023).

Major comments:

1. On page 2 (lines 74-76), the authors highlight a key unavoidable limitation of their proposed work, where the variety of ototoxicity scales "complicates the comparison of hearing loss outcome". The authors propose adopting a dichotomous classification of ototoxicity to account for this limitation. While this is likely the only way that authors would be able to compare non-equivalent outcomes, differences in study designs means that grade 1 ototoxicity (or mild ototoxicity) has been collapsed with no ototoxicity, considered as ototoxicity and/or completely excluded from comparisons. Therefore, the distinctions between ototoxicity and no ototoxicity will be highly heterogeneous when combining results between studies unless new groupings can be made based on published data. It is important to better highlight this limitation. The authors should also refer to a previously published paper that compared ototoxicity classification schemes and graded patients across scales to look at concordance (Clemens E, Brooks B, de Vries ACH, van Grotel M, van den Heuvel-Eibrink MM, Carleton B. A comparison of the Muenster, SIOP Boston, Brock, Chang and CTCAEv4.03 ototoxicity grading scales applied to 3,799 audiograms of childhood cancer patients treated with platinum-based chemotherapy. PLoS One. 2019 Feb 14;14(2):e0210646. doi: 10.1371/journal.pone.0210646. PMID: 30763334).
2. On page 3 (lines 122-125), the authors propose capturing all variant associations (i.e., both significant and non-significant associations) for candidate genes studies but only significant associations for GWAS or large-scale candidate genes studies. The rationale for including only Bonferroni-corrected GWAS significant results but including nonsignificant variants from candidate gene studies with their inherent limitations is unclear.
3. On page 3 (lines 126-127), the authors propose performing meta-analyses for any variants that have been reported in more than one study with reasonably similar study populations, but it is unclear what would count as "reasonably similar", especially in relation to proposed sensitivity analyses later in the protocol that would attempt to look at sources of heterogeneity between studies/populations.
4. On page 3 (line 128), it is unclear how the authors propose reporting on co-factors involved in platinum-induced hearing loss. Specifically, do the authors plan to report only those that are significantly associated ($P<0.05$)? It is important to note whether any statistical associations were conducted/reported for non-genetic factors since this is not always done. We would also caution that platinum cumulative dose is not a good indicator of hearing loss propensity because serial audiometric monitoring shows that hearing loss occurs and plateaus well before cisplatin treatment is completed. See Siemens A, Brooks B, Rassekh SR, Meijer AJM, van den Heuvel-Eibrink MM, Xu W, Loucks CM, Ross CJD, Carleton BC; Canadian Pharmacogenomics Network for Drug Safety Consortium. Role of Cisplatin Dose Intensity and TPMT Variation in the Development of Hearing Loss in Children. Ther Drug Monit. 2023 Jun 1;45(3):345-353. doi: 10.1097/FTD.0000000000001085. PMID: 36917731.

5. On page 3 (lines 131-132), the newer version of PRISMA guidelines should be followed and cited.
6. On page 3 (lines 167-168), the authors again say they will only report variants that have significant associations from large candidate gene studies and GWAS. However, the authors have not indicated how significance is defined. For example, will they consider only the GWAS Bonferroni-corrected significance threshold of $5x10^{-8}$? Any other threshold should be justified and please again refer to the question in point #2 above about the inclusion of all variants from candidate gene studies.
7. On page 4 (line 176), will older versions of CTCAE grading be considered? If not, several relevant studies will not be included. It is also important to recognize that the inclusion of Grade 1 hearing loss is subclinical in that it does not interfere with day-to-day communication. Genetic variants that are associated with this degree (and only this degree) of hearing loss are unlikely to be clinically relevant. The other problem with inclusion of grade 1 is that cisplatin is a heavy metal and as such, hearing loss is progressive over time despite discontinuation. Someone who has grade 1 hearing loss may not always remain at grade 1. Grade 2 and above are thresholds that are clinically actionable, at least through intensified monitoring.
8. On page 4 (lines 206-207), independent data extraction and checking should be conducted given that a second reviewer may be swayed by the first reviewer's extracted data.
9. On page 5 (quality/validity assessment sections), it is unclear how the various scales/approaches will be integrated. It is also unclear why a single tool designed for genetic association studies was not chosen (e.g., Q-Genie informed by guidelines and recommendations on appropriate conduct of genetic association studies such as STREGA guidelines). In particular, assuring alignment with STREGA guidelines is important, but other study-related quality aspects (apart from genotyping quality) could also be assessed using STREGA guidelines (or tools like Q-Genie).
10. On page 6 (lines 288-290), it is unclear how the authors would consider the potential bias that negative genetic association results are less likely to be published when interpreting their results.

Minor comments:

1. On page 6 (line 288), the authors likely mean "inherent" and not "inherited".

VERSION 1 - AUTHOR RESPONSE

Reviewer comments to authors:

In this work, the authors provide a detailed protocol that will specifically quantify the impact of genetic variants on the likelihood of developing platinum-induced ototoxicity in children. The authors should leverage from some of the recent work of previous systematic reviews. For example, a recent systematic review conducted by our group (Scott, E. N. *et al.* Systematic Critical Review of Genetic Factors Associated with Cisplatin-induced Ototoxicity: Canadian Pharmacogenomics Network for Drug Safety 2022 Update. *Ther Drug Monit* (2023) doi:10.1097/FTD.0000000000001113) was cited by the authors primarily to highlight that a similar review to that proposed has not been conducted (our review included both children and adults). However, our review would have included most, if not all, of the relevant paediatric studies (up to June 2020) with the caveat that we did not consider carboplatin-specific studies (but these studies are likely to be few if any since carboplatin is mostly used after cisplatin has already been provided). Comparing paediatric studies retrieved in our study to those retrieved by their search strategy will help determine what the new study will provide compared to the existing one.

We thank both reviewers for this important comment. We agree that the recent systematic review published by Scott E. N. *et al.* likely includes most of the association studies on gene variants and PIHL in children published before June 2020. However, at least three major studies [1–3] have been published since then and contribute meaningfully to our understanding of genotype–phenotype associations in the paediatric population.

We modified the introduction paragraph as follows: "Although the most recent systematic review was published in 2023 [23], it does not include key paediatric studies published after June 2020 [10,28,29], underscoring the need for an updated review focused on children." (page 2, lines 111-113)

Furthermore, by focusing exclusively on children, our review will exclude findings that are not relevant to this population—such as variants that may only emerge as significant in ageing individuals—and may highlight genetic variants particularly relevant to paediatric patients, whose effects could otherwise be diluted in mixed paediatric–adult analyses. This argument was already addressed in the discussion paragraph (page 6, lines 311-313).

Furthermore, while study quality should be assessed, our prior study already assessed the quality of several studies likely to be included in their work using a tool designed for genetic association studies (Sohani, Z. N. *et al.* Assessing the quality of published genetic association studies in meta-analyses: the quality of genetic studies (Q-Genie) tool. *BMC Genet* 16, 50 (2015)).

We address this point below in Major Comments point 9.

Finally, it is likely that the combined impact for many variants will lead to a relatively modest effect size (especially given the inherent heterogeneity between studies). Therefore, it is important to provide more rationale on the value of quantifying the magnitudes of effect (when many are likely to be small). For example, in our prior review, we chose to highlight only variants that had been associated with ototoxicity in at least three independent patient populations with magnitudes of effect (i.e., odds ratios) greater than 3 (or less than0.33) due to the clinical importance of such strong effect sizes.

We agree that modest effect sizes are to be expected, particularly given the limited number of paediatric studies available and the inter-study heterogeneity. While we fully acknowledge the clinical relevance of large effect sizes (e.g., OR > 3 or < 0.33), we also believe it is important to report and interpret more modest associations. This is particularly relevant in the context of PIHL, a complex and likely polygenic trait, where cumulative genetic risk—rather than the effect of a single variant—may be more informative. Moreover, considering the irreversible nature of PIHL and its significant impact on language development in children, even modest associations may hold clinical value.

We will therefore report all pooled odds ratios, regardless of their magnitude, and will carefully interpret the strength of each association based on its effect size, confidence interval width, p-value, and robustness in sensitivity analyses. This approach allows for a comprehensive and nuanced understanding of the genetic architecture underlying PIHL.

We modified the discussion paragraph as follows: "Given the polygenic nature of PIHL, all pooled odds ratios will be reported, including modest associations." (page 6, lines 335-336)

Given that published manuscripts differ with respect to publishing requirements, it is essential not to compare "published studies" but populations of patients to determine if findings have been replicated. Nature Genetics for example requires two independent

populations and biological validation. This is quite a bit more stringent that many other journals.

We fully agree that replication should be assessed at the level of independent study populations, rather than at the level of published articles. We will therefore consider the results from discovery and validation cohorts within the same publication separately, as long as they represent truly independent populations, and the replication cohort is not already included in another study.

We have replaced the statement “published studies” with “independent study populations” where appropriate throughout the manuscript (page 3, line 124; page 4, line 149, 184; page 5, lines 255, 264, 274; page 6, lines 329).

Overall, while the protocol for this proposed review is well-described, it is unclear whether this review will net much new information since this topic has already been reviewed recently (2023).

We addressed this comment above, in “Global Remarks”, point 1.

Major comments:

1. On page 2 (lines 74-76), the authors highlight a key unavoidable limitation of their proposed work, where the variety of ototoxicity scales “complicates the comparison of hearing loss outcome”. The authors propose adopting a dichotomous classification of ototoxicity to account for this limitation. While this is likely the only way that authors would be able to compare non-equivalent outcomes, differences in study designs means that grade 1 ototoxicity (or mild ototoxicity) has been collapsed with no ototoxicity, considered as ototoxicity and/or completely excluded from comparisons. Therefore, the distinctions between ototoxicity and no ototoxicity will be highly heterogeneous when combining results between studies unless new groupings can be made based on published data. It is important to better highlight this limitation. The authors should also refer to a previously published paper that compared ototoxicity classification schemes and graded patients across scales to look at concordance (Clemens E, Brooks B, de Vries ACH, van Grotel M, van den Heuvel-Eibrink MM, Carleton B. A comparison of the Muenster, SIOP Boston, Brock, Chang and CTCAEv4.03 ototoxicity grading scales applied to 12. 3. 4. 5. 6. 7. 8. 9. 3,799 audiograms of childhood cancer patients treated with platinum-based chemotherapy. PLoS One. 2019 Feb 14;14(2):e0210646. doi: 10.1371/journal.pone.0210646. PMID: 30763334).

   We thank the reviewer for this insightful comment. We have clarified our methodology to specify that hearing loss will be extracted as a dichotomous outcome (yes/no) based on how it is reported in each independent study population. We agree that the heterogeneous classification of Grade 1 ototoxicity—sometimes considered as no ototoxicity, as ototoxicity, or excluded altogether—poses a major challenge to comparability.

   To address this, we have expanded our discussion of this limitation and will conduct multiple sensitivity analyses stratified by how Grade 1 was handled:

   - G0 vs. G≥1
   - G0–1 vs. G≥2
   - G0 vs. G≥2 (excluding G1)

   As individual-level audiogram data are rarely available, systematic reclassification by ototoxicity grade will not be feasible. However, when data allow, we will recategorise

outcomes based on common grading schemes to assess the impact of Grade 1 classification and evaluate robustness.

Additionally, in studies reporting hearing loss solely as hearing aid use—a proxy for SIOP grade ≥3—we will include this outcome and conduct a separate sensitivity analysis (e.g., G0–2 vs. G≥3).

Finally, we now cite Clemens et al. (PLoS One, 2019; PMID: 30763334), who reported moderate to high concordance between grading scales (e.g., 0.636 for Chang vs. Muenster and 0.975 for Brock vs. Chang), while acknowledging residual inconsistencies, particularly for early grades.

We modified the strengths and limitations paragraph as follows: "Variability in ototoxicity grading scales limits comparability across studies; to address this, we will apply a dichotomous classification (hearing loss: yes/no) and perform sensitivity analyses by severity and grading system where feasible." (page 2, lines 75-77).

We modified the eligibility criteria; outcome paragraph as follows: "Various ototoxicity grading systems are used in children such as ASHA [33], CTCAE (version 5.0 or earlier) [34–38], Brock, Chang, Muenster, or SIOP Boston scales (Table 2) [33,39,40]. Moreover, various classification schemes are used across studies to distinguish between hearing loss and no hearing loss (e.g., G0 vs. G≥1; G0–1 vs. G≥2; G0 vs. G≥2 excluding G1), with Grade 1 being the most heterogeneously classified. In this study, the outcome hearing loss will be dichotomized as present or absent (yes/no), as reported in each independent study population. We will include studies reporting hearing loss based on recognised ototoxicity grading scales (Table 2) or based on the reported use of hearing aids after treatment when no formal grading is available." (page 4, lines 178-185)

We modified the data synthesis and re-analysis paragraph as follows:
"For each genetic variant, the number of patients with and without hearing loss will be extracted— as reported in each independent study population—and we will include its reported univariate analysis results [odds ratio (OR), 95% confidence interval (CI), P-value]." (page 5, lines 254-257)
"For independent study populations in which the exact number of patients per ototoxicity grade is available, we will recategorize the data according to the most common classification schemes used in the meta-analysis (e.g., G0 vs. G≥1; G0–1 vs. G≥2; G0 vs. G≥2 excluding G1) to maximise comparability." (page 5, lines 264-266)

We modified the robustness assessment paragraph as follows: "Sensitivity analyses will be conducted whenever possible to assess the robustness of our findings, including stratification by study quality, presence of non-genetic covariates (e.g., age at treatment, cranial radiation, platinum exposure, concomitant ototoxic drugs) and handling of Grade 1 ototoxicity (e.g., G0 vs. G≥1; G0–1 vs. G≥2; G0 vs. G≥2 excluding G1), and use of hearing aids (e.g., G0–2 vs. G≥3). If data allow, analyses stratified by grading system will also be conducted." (page 6, lines 288-292)

We modified discussion paragraph as follows: "To enable meta-analysis, we chose to dichotomize hearing loss as present or absent. However, this approach may introduce substantial heterogeneity due to differences between grading scales and the inconsistent handling of Grade 1, which is particularly problematic given its variable clinical relevance and potential to worsen over time. Although overall concordance between grading systems is generally good [34], discrepancies persist, especially in early grades. To address this, we will assess heterogeneity using the I^2 index and conduct sensitivity analyses. Finally, to avoid excluding studies that report hearing loss

only through hearing aids use, we will consider hearing aids use as a proxy for severe ototoxicity (G≥3) [34].” (page 7, lines 347-354)

The suggested reference Clemens E. et al. (2019) was already included in the manuscript, but it is now discussed in more detail in the discussion paragraph, as mentioned above.

2. On page 3 (lines 122-125), the authors propose capturing all variant associations (i.e., both significant and non-significant associations) for candidate genes studies but only significant associations for GWAS or large-scale candidate genes studies. The rationale for including only Bonferroni-corrected GWAS significant results but including nonsignificant variants from candidate gene studies with their inherent limitations is unclear.

We thank the reviewers for this important and constructive comment. We agree that clarification was needed regarding both our significance thresholds and the rationale for our approach to selecting variant associations across different study designs.

For hypothesis-driven candidate gene studies, we have reconsidered our strategy regarding the inclusion of non-significant variants. We will initially include all variants reported as significantly associated, using a p-value threshold of <0.05.

For GWAS and large-scale candidate gene studies (i.e., those testing >100 variants), we will also include all variants reported as statistically significant using author defined multiple testing correction. If no multiple test correction is provided, we will apply Bonferroni correction, or the conventional genome-wide threshold ($p < 5\times10^{-8}$) for GWAS. In addition, we will consider reporting variants that reach the commonly used threshold for suggestive significance ($p < 1 \times 10^{-5}$) in large-scale studies that do not report any findings meeting the $p < 5 \times 10^{-8}$ threshold [1,2].

We will generate our initial list based only on statistically significant associations reported in at least one candidate gene studies or GWAS. For each of these variants, we will then extract their corresponding association results from all other included studies, regardless of significance. This approach ensures that previously identified variants of interest are consistently evaluated across available datasets.

We modified the aims and objectives paragraph as follows: “• Collecting and analysing genetic variants tested for an association with PIHL in children, including: (i) all variants reported as significantly associated in at least one candidate gene study ($p < 0.05$) or genome-wide association studies (GWAS) and large-scale candidate gene study (using author-defined correction, or Bonferroni correction or $p < 5\times10^{-8}$ if not specified); and (ii) the corresponding association results—regardless of significance—from all other independent study populations analysing the same variant.” (page 3, lines 119-124).

We modified the eligibility criteria; comparison paragraph as follows: “We will include all variants significantly associated with PIHL in at least one candidate gene study or GWAS, using an adjusted p-value <0.05. Significance will be defined using author defined multiple testing correction. If no multiple test correction is provided, we will apply Bonferroni correction, or the conventional genome-wide threshold ($p < 5\times10^{-8}$) for GWAS. For each included variant, we will extract association results—regardless of significance—from all other independent study populations where the same variant was analysed. If a large scale study (>100 gene variants tested) does not

report genome-wide significant findings, we will consider using variants meeting the suggestive threshold ($p < 1\times10^{-5}$)." (page 3-4, lines 165-172)

We added a paragraph in the discussion as follows: "To evaluate reproducibility, we will identify variants initially reported as significant in at least one independent study population and examine their association results in other included datasets analysing the same variant, regardless of significance. This approach facilitates cross-validation of previously reported associations across diverse study populations, though we acknowledge a potential risk of selection bias." (page 6, lines 328-332)

3. On page 3 (lines 126-127), the authors propose performing meta-analyses for any variants that have been reported in more than one study with reasonably similar study populations, but it is unclear what would count as "reasonably similar", especially in relation to proposed sensitivity analyses later in the protocol that would attempt to look at sources of heterogeneity between studies/populations.

   We agree that the phrase "reasonably similar" was unclear. We have removed it from the paragraph on aims and objectives, while retaining the proposed sensitivity analyses later in the protocol.

   We modified the aims and objectives paragraph as follows: "Performing a meta-analysis of all variants reported as significant in more than one independent paediatric study population." (page 3, lines 125-126)

   We modified the data analysis paragraph as follows: "Each genetic variant that has been reported as significantly associated with PIHL in more than one independent study population will be considered for the meta-analysis." (page 5, lines 337-338)

4. On page 3 (line 128), it is unclear how the authors propose reporting on co-factors involved in platinum-induced hearing loss. Specifically, do the authors plan to report only those that are significantly associated ($P<0.05$)? It is important to note whether any statistical associations were conducted/reported for non-genetic factors since this is not always done. We would also caution that platinum cumulative dose is not a good indicator of hearing loss propensity because serial audiometric monitoring shows that hearing loss occurs and plateaus well before cisplatin treatment is completed. See Siemens A, Brooks B, Rassekh SR, Meijer AJM, van den Heuvel-Eibrink MM, Xu W, Loucks CM, Ross CJD, Carleton BC; Canadian Pharmacogenomics Network for Drug Safety Consortium. Role of Cisplatin Dose Intensity and TPMT Variation in the Development of Hearing Loss in Children. Ther Drug Monit. 2023 Jun 1;45(3):345-353. doi:10.1097/FTD.0000000000001085. PMID: 36917731.

   We thank the reviewer for this valuable comment and the reference provided. In response, we clarify that we will narratively report all non-genetic covariates investigated in the included studies (e.g. age at treatment, cranial radiation, platinum exposure, concomitant ototoxic drugs), whether statistical testing was performed and whether statistically significant associations were observed.

   We modified the strengths and limitations paragraph as follows: "All clinical covariates influencing the risk of PIHL will be extracted from each study and reported narratively in the data synthesis. Sensitivity analysis will be conducted to assess the robustness of findings." (page 2, lines 88-91)

   We modified the aims and objectives paragraph as follows: "All clinical covariates related to PIHL risk will be narratively summarised, with sensitivity analyses conducted to assess the robustness of results." (page 3, lines 78-79)

We modified the eligibility criteria; outcomes paragraph as follows: “The secondary outcome includes the association of non-genetic covariates with PIHL, such as age at treatment, details of the platinum regimen, cranial radiation exposure, and exposure to other ototoxic drugs. For each covariate, we will report whether statistical testing was performed and whether a statistically significant association was observed.” (page 4, lines 187-190)

We modified data synthesis and re-analysis paragraph as follows: “Non-genetic covariates will be reported narratively, including whether they were assessed statistically. Where available, we will report subgroup or stratified odds ratios as reported in the original studies, to explore potential effect modification by clinical covariates (e.g., age at treatment, cranial radiation, platinum exposure, concomitant ototoxic drugs).” (page 5, lines 258-261)

Regarding platinum exposure, we acknowledge the limitations of cumulative dose as a proxy for ototoxic risk, as highlighted by Siemens *et al.* (2023). we will extract reported indicators of cisplatin treatment intensity—such as daily dose, number of cycles, and dosing intervals—as these may better reflect ototoxic risk, as suggested in the referenced study. We will also report whether statistical testing was performed and whether statistically significant associations were observed.

We modified the introduction paragraph as follows: “Known clinical risk factors for PIHL are the use of cisplatin compared to carboplatin, higher cisplatin dose intensity, younger age at exposure, concomitant cranial radiation, renal insufficiency and administration of other ototoxic drugs (e.g. aminoglycoside antibiotics, furosemide loop diuretics and vincristine) [10,17–19].” (page 2, lines 103-106)

We modified the eligibility criteria; outcomes paragraph as follows: “PIHL is defined as bilateral, often permanent sensorineural hearing loss, primarily affecting high frequencies and worsening with platinum dose intensity [14,31,32].” (page 4, lines176-178)

We modified the data collection and extraction paragraph as follows: “[…] covariates and their inclusion in multivariable models [age at time of platinum treatment, cumulative dose of platinum compound received (mg/m2), maximum dose of platinum compound (mg/m2) per day, number of cycles of platinum compound, dosing intervals, […]” (page 5, lines 229-231)

The suggested reference Siemens et al. (2023) was already included in the manuscript.

5. On page 3 (lines 131-132), the newer version of PRISMA guidelines should be followed and cited.
   We thank the reviewers for noting that the PRISMA-P 2015 guidelines were cited instead of the updated PRISMA 2020.

   We have now cited the PRISMA 2020 guidelines and will adhere to them for the reporting of the final systematic review and meta-analysis (page 3, lines 130-131):
   - Page MJ, McKenzie JE, Bossuyt PM, et al. The PRISMA 2020 statement: an updated guideline for reporting systematic reviews. BMJ. 2021;372:n71. doi: 10.1136/bmj.n71

   However, as PRISMA 2020 is not intended to guide the reporting of protocols, we have retained the PRISMA for Protocols (PRISMA-P) 2015 checklist to ensure that the

current protocol is complete and complies with established standards for protocol reporting. We ensured that no additional methodological items required by the PRISMA 2020 guidelines were omitted, and that all relevant elements are covered by the PRISMA-P 2015 framework.

We modified the discussion paragraph as follows: “This protocol details the methodology of our systematic review and meta-analysis, aiming to summarize current knowledge on genetic variants influencing PIHL risk in children and fulfils all requirements of the PRISMA-P 2015 checklist for protocols (Supplemental table 2) [46].” (page 6, lines 309-311)

6. On page 3 (lines 167-168), the authors again say they will only report variants that have significant associations from large candidate gene studies and GWAS. However, the authors have not indicated how significance is defined. For example, will they consider only the GWAS Bonferroni-corrected significance threshold of 5x10-8? Any other threshold should be justified and please again refer to the question in point #2 above about the inclusion of all variants from candidate gene studies.

   We address this point above in Major Comments point 2.

7. On page 4 (line 176), will older versions of CTCAE grading be considered? If not, several relevant studies will not be included. It is also important to recognize that the inclusion of Grade 1 hearing loss is subclinical in that it does not interfere with day-to-day communication. Genetic variants that are associated with this degree (and only this degree) of hearing loss are unlikely to be clinically relevant. The other problem with inclusion of grade 1 is that cisplatin is a heavy metal and as such, hearing loss is progressive over time despite discontinuation. Someone who has grade 1 hearing loss may not always remain at grade 1. Grade 2 and above are thresholds that are clinically actionable, at least through intensified monitoring.

We thank the reviewer for this thoughtful and important comment. We confirm that older versions of the CTCAE grading system will be included in our analysis, although this was not clearly stated in the original manuscript. We have revised the text to clarify this point. In addition, because some studies report ototoxicity solely in terms of the need for hearing aids following chemotherapy, we will also include this functional endpoint to avoid excluding relevant studies. To address the concern about the progressive nature of cisplatin-induced hearing loss, we will collect and report follow-up time to hearing assessments in each independent patient population, where available. This will allow us to account for the potential evolution of hearing loss severity over time.

We added the following additional references in Table 2 and page 4 lines 218-228:

- Common Terminology Criteria for Adverse Events (CTCAE) v4.03.
- Common Terminology Criteria for Adverse Events (CTCAE)v4.0. 2009.
- Trotti A, Colevas AD, Setser A, *et al.* CTCAE v3.0: development of a comprehensive grading system for the adverse effects of cancer treatment. *Semin Radiat Oncol*. 2003;13:176–81. doi: 10.1016/S1053-4296(03)00031-6
- Trotti A, Byhardt R, Stetz J, *et al.* Common toxicity criteria: version 2.0. an improved reference for grading the acute effects of cancer treatment: impact on radiotherapy. *Int J Radiat Oncol Biol Phys*. 2000;47:13–47. doi: 10.1016/s0360-3016(99)00559-3

Table 2 was adapted accordingly to reflect these adjustments.

We modified the eligibility criteria; outcome paragraph as follows: "Various ototoxicity grading systems are used in children such as ASHA [33], CTCAE (version 5.0 or earlier) [34–38], Brock, Chang, Muenster, or SIOP Boston scales (Table 2) [33,39,40]." (page 4, lines 178-180)

We modified the data collection and extraction paragraph as follows: "[…] tinnitus and vertigo symptoms, length of follow-up to outcome measurement, data metrics and statistical analysis […]" (page 4, lines 227-228)

The limitations associated with grade 1 hearing loss have been addressed above under Major Comments, point 1.

8. On page 4 (lines 206-207), independent data extraction and checking should be conducted given that a second reviewer may be swayed by the first reviewer's extracted data.

We thank the reviewers for this important comment. We agree that the optimal approach to minimize bias in data extraction is to have two independent reviewers extract data separately. However, due to resource limitations, we will be unable to conduct fully independent double extraction for all included studies—estimated to range between 30 and 40 based on previous systematic reviews [4,5]. Instead, data will be extracted by one reviewer and checked by a second, with any discrepancies resolved by consensus or adjudication by a third reviewer. The third reviewer will be an expert of the category of data extracted for genetics and clinical data.

We modify the data collection and extraction paragraph as follows: "Full reports of all included manuscripts will be examined for data extraction by a first reviewer. A second reviewer will check the extracted data. Any disagreements will be resolved by a third reviewer with expertise in the relevant domain (e.g., genetics, clinical oncology)." (page 4, lines 217-219)

9. On page 5 (quality/validity assessment sections), it is unclear how the various scales/approaches will be integrated. It is also unclear why a single tool designed for genetic association studies was not chosen (e.g., Q-Genie informed by guidelines and recommendations on appropriate conduct of genetic association studies such as STREGA guidelines). In particular, assuring alignment with STREGA guidelines is important, but other study-related quality aspects (apart from genotyping quality) could also be assessed using STREGA guidelines (or tools like Q-Genie).

We thank the reviewers for this important clarification. As our study focuses exclusively on genetic association studies, we agree that the Q-Genie tool is the most appropriate instrument to assess methodological quality. In response, we have removed the ROBINS-E and CASP tools from our protocol and clarified that Q-Genie will be used as the sole quality and validity assessment tool. The STREGA guidelines will be used as complement to define useful genotyping quality details that are not explicitly covered by the Q-Genie tool.

We modify the data collection and extraction; quality and validity assessment paragraph as follows: "Two reviewers will independently assess the quality of each included study using the Q-Genie tool, a validated instrument for genetic association studies [42]. In case of disagreement, a third reviewer will resolve the conflict. Genotyping quality metrics—such as sequencing method, call-rates, genotyping procedure, Hardy-Weinberg Equilibrium and multiple test corrections (e.g., Bonferroni,

False Discovery Rate)—will be reported descriptively to complement the Q-Genie assessment and ensure alignment with STREGA recommendations, on which Q-Genie is partly based [43]. A global validity and quality assessment statement will be issued for each study." (page 5, lines 242-248).

We added the following suggested reference (page 5, line 288):

- Sohani ZN, Meyre D, de Souza RJ, et al. Assessing the quality of published genetic association studies in meta-analyses: the quality of genetic studies (Q-Genie) tool. BMC Genet. 2015;16:50. doi: 10.1186/s12863-015-0211-2

We modified the discussion paragraph as follows: "The Q-Genie tool [42] will be applied to all included studies. For those previously assessed with Q-Genie [23], we will compare scores to ensure consistency and maintain methodological rigour across the evidence base." (page 6, line 338-341)

10. On page 6 (lines 288-290), it is unclear how the authors would consider the potential bias that negative genetic association results are less likely to be published when interpreting their results.

We thank the reviewers for raising this important point. We fully acknowledge the potential for publication bias, particularly the underreporting of non-significant genetic associations in the published literature. As this bias cannot be formally corrected in the context of our review, we will address it qualitatively in the discussion. This limitation will be clearly acknowledged when interpreting our findings and assessing the strength of the evidence.

We modified the discussion paragraph as follow: "Formal assessment of publication bias (e.g., Funnel plot, Egger's test) is unlikely due to the limited number of studies per variant, but potential bias—including underreporting of non-significant findings—will be considered." (page 6, lines 336-338)

Minor comments:

1. On page 6 (line 288), the authors likely mean "inherent" and not "inherited".
   We replaced "inherited" by "inherent" (page 6, line 373).

Additional modification:

We would like to inform you that we identified a missing institutional affiliation for Brice Fresneau in the original submission. We regret this omission and thank you for your understanding.

We have updated the manuscript accordingly to reflect the correct information: "[5] Gustave Roussy, Department of Children and Adolescents Oncology, Villejuif, F-94805, France; [6] Inserm U1018 CESP, Radiation Epidemiology team, Paris-Saclay University, Gustave Roussy Cancer Campus, Villejuif, France" (page 1, lines 16-18).

References

1 Siemens A, Brooks B, Rassekh SR, *et al.* Role of Cisplatin Dose Intensity and TPMT Variation in the Development of Hearing Loss in Children. *Therapeutic Drug Monitoring*. 2023;45:345. doi: 10.1097/FTD.0000000000001085
2 Meijer AJM, Li KH, Brooks B, *et al.* The cumulative incidence of cisplatin-induced hearing loss in young children is higher and develops at an early stage during therapy compared with older children based on 2052 audiological assessments. *Cancer*. 2022;128:169–79. doi: 10.1002/cncr.33848
3 Hurkmans EGE, Klumpers MJ, Dello Russo C, *et al.* Genome-wide analyses of platinum-induced ototoxicity in childhood cancer patients: Results of GO-CAT and United Kingdom MAGIC consortia. *Front Pharmacol*. 2022;13:980309. doi: 10.3389/fphar.2022.980309
4 Scott EN, Joseph AA, Dhanda A, *et al.* Systematic Critical Review of Genetic Factors Associated with Cisplatin-induced Ototoxicity: Canadian Pharmacogenomics Network for Drug Safety 2022 Update. *Ther Drug Monit*. 2023;45:714–30. doi: 10.1097/FTD.0000000000001113
5 Tserga E, Nandwani T, Edvall NK, *et al.* The genetic vulnerability to cisplatin ototoxicity: a systematic review. *Sci Rep*. 2019;9:3455. doi: 10.1038/s41598-019-40138-z

---

VERSION 2 - REVIEW

**Reviewer**	**1**
**Name**	**Carleton, Bruce**
**Affiliation**	**University of British Columbia**
**Date**	**29-Jul-2025**
**COI**	

We thank the authors for the thoughtful responses to our comments. We would like to re-state that the protocol for this proposed review is well-described; however, the revised protocol makes it clear that this review will not net much new information since this topic has already been reviewed recently (2023) and only three pediatric studies have been published since then. Therefore, it is recommended that the authors adjust the focus/rationale of their protocol compared to the prior review. For example, this review takes a quantitative approach to detect small/moderate effects that could help better elucidate the polygenic nature of cisplatin-induced ototoxicity.<p>

Major comments:<p>

1. It is recommended that the authors adjust this new sentence: “Although the most recent systematic review was published in 2023 [23], it does not include key paediatric studies published after June 2020 [10,28,29], underscoring the need for an updated review focused on children.” Reference 10 focuses on how a known genetic association is affected by cisplatin dose intensity and references 28/29 identify two new GWAS signals. Therefore, there are not a lot of new genetic associations to review. However, these additions, coupled with the more quantitative approach of the proposed work, could make the rationale for this review more compelling.<p>

2. It is unclear why the authors would want to contaminate their control (nonototoxicity) groups by including grade 1 ototoxicity in this group. The authors stated that they would conduct sensitivity analyses: Gr0 v. Gr1 or higher; Gr0 v. Gr2 or higher and Gr0-1v. Gr2 or higher. However, combining Gr0 (no ototoxicity) with Gr1 (mild ototoxicity) only contaminates the control group. The authors later state another analysis is Gr0-2 v. Gr3 or higher. This also creates problems as the authors are grouping in no ototoxicity with patients that have ototoxicity. Since the authors are trying to detect variants that relate to ototoxicity, this is a confusing sensitivity analysis. It is recommended that the authors consider a primary analysis with grade 1 cases not included in the control group. A major goal of this work should be to promote the use of clear case/control categories for future studies, so that the quality of cisplatin-induced ototoxicity genetic association studies continues to improve.<p>

3. If the authors want to look at variants based on grade of hearing loss, they will need to do a dose and dose intensity exposure analysis (NOT cumulative dose exposure) since the amount of cisplatin exposure is important in concentration-dependent adverse reactions. Cumulative exposure is not relevant because hearing loss occurs and stabilizes before cumulative exposure is reached. The lack of serial audiograms for many European centres means they use cumulative exposure but this is not a valid surrogate for when and the degree of hearing loss as previous papers have noted. It is recommended that the authors include this as a major limitation in their protocol (and most cisplatin-induced ototoxicity genetic association studies).<p>

Minor comments:<p>

1. CTCAE has no "grade 0" group. Those who have no ototoxicity are classified as controls. Ototoxicity and other CTCAE adverse events start with Grade 1 and continue up from there. Please adjust this in the manuscript.

VERSION 2 - AUTHOR RESPONSE

Comments to the Author:

We thank the authors for the thoughtful responses to our comments. We would like to re-state that the protocol for this proposed review is well-described; however, the revised protocol makes it clear that this review will not net much new information since this topic has already been reviewed recently (2023) and only three paediatric studies have been published since then. Therefore, it is recommended that the authors adjust the focus/rationale of their protocol compared to the prior review. For example, this review takes a quantitative approach to detect small/moderate effects that could help better elucidate the polygenic nature of cisplatin-induced ototoxicity.

We thank the reviewer for his encouraging comment, and we address the focus/rationale point in major comments 1.

Major comments:

1. It is recommended that the authors adjust this new sentence: “Although the most recent systematic review was published in 2023 [23], it does not include key paediatric studies published after June 2020 [10,28,29], underscoring the need for an updated review focused on children.” Reference 10 focuses on how a known genetic association is affected by cisplatin dose intensity and references 28/29 identify two new GWAS signals. Therefore, there are not a lot of new genetic associations to review. However, these additions, coupled with the more quantitative approach of the proposed work, could make the rationale for this review more compelling.

We thank the reviewer for this helpful and constructive suggestion. We agree that only a few additional paediatric studies have been published since the 2023 systematic review [23], and we acknowledge that the volume of new data alone is limited. In response, we have revised the rationale of our protocol to emphasize its added value beyond the inclusion of recent studies. Specifically, our review focuses exclusively on children, which might uncover genetic effects specific to this population, and adopts a quantitative approach, which aims to detect small to moderate genetic effects that may be overlooked in individual studies. This approach may help clarify the polygenic nature of cisplatin-induced ototoxicity and contribute to a better understanding of children-specific susceptibility.

We modified the abstract introduction paragraph as follows: “Since genetic susceptibility in children may differ from adults, and given the critical window of auditory development, a focused investigation of paediatric genetic factors using quantitative methods is warranted to detect small to moderate effects and understand the polygenic nature of PIHL.” (page 1, lines 45-48)

We modified the strengths and limitations paragraph as follows: “The strength of this systematic review and meta-analysis is its exclusive focus on the paediatric population and its quantitative approach, which may help uncover small to moderate genetic effects contributing to the polygenic nature of platinum-induced hearing loss in a patient population with specific characteristics.” (page 2, lines 71-75)

We modified the introduction paragraph as follows: “Genetic predispositions likely contribute to this variability, and the inconsistent findings across studies may reflect modest individual effects within a broader polygenic framework [20–22]. Previous systematic reviews have combined adult and paediatric populations [23–26], with only one 2017 meta-analysis focusing exclusively on children, limited to TPMT, COMT, and ACYP2 genes [27]. The most recent systematic review was published in 2023 [23] and offered key insights into genetic susceptibility to PIHL across age groups. While only a few additional paediatric studies have emerged [10,28,29], this review builds on previous work by focusing specifically on children and taking a quantitative approach to detect small to moderate effects and clarify its polygenic nature.” (page 2-3, lines 109-116)

We modified the aims and objectives paragraph as follows: “Performing a meta-analysis of all variants reported as significant in more than one independent paediatric study population, to enable detection of small to moderate effects and clarify the polygenic nature of PIHL.” (page 3, lines 128-130)

We modified the discussion paragraph as follows: “By focusing on the paediatric population and taking a quantitative approach, this review addresses the possibility of unique genetic ototoxicity risk variants in this group that may not have been detected in studies combining adult and paediatric populations. Additionally, the review may help detect small to moderate effects, clarify the polygenic nature of PIHL.” (page 6, lines 315-319)

2. It is unclear why the authors would want to contaminate their control (nonototoxicity) groups by including grade 1 ototoxicity in this group. The authors stated that they would conduct

sensitivity analyses: Gr0 v. Gr1 or higher; Gr0 v. Gr2 or higher and Gr0-1v. Gr2 or higher. However, combining Gr0 (no ototoxicity) with Gr1 (mild ototoxicity) only contaminates the control group. The authors later state another analysis is Gr0-2 v. Gr3 or higher. This also creates problems as the authors are grouping in no ototoxicity with patients that have ototoxicity. Since the authors are trying to detect variants that relate to ototoxicity, this is a confusing sensitivity analysis. It is recommended that the authors consider a primary analysis with grade 1 cases not included in the control group. A major goal of this work should be to promote the use of clear case/control categories for future studies, so that the quality of cisplatin-induced ototoxicity genetic association studies continues to improve.

We thank the reviewer for raising this important point regarding phenotype definition and case/control classification. We fully agree that including individuals with grade 1 ototoxicity in the control group can introduce misclassification and reduce the contrast needed to detect genetic associations. However, ototoxicity grading systems and classification thresholds vary widely across studies, and in some studies grade 1 was already included among controls.
To avoid excluding a large portion of the available data, our primary analysis will follow the dichotomization reported in each study (hearing loss: yes/no), as originally defined by the authors. This inclusive approach maximizes power, but we acknowledge it introduces heterogeneity—for instance, by pooling studies using a G0 vs. G≥1 scheme with others using G0–1 vs. G≥2. When feasible—e.g., when the distribution of hearing loss grades is provided—we will reclassify studies that did not originally use a comparable classification scheme, to reduce heterogeneity while retaining a maximal power.
In a second time, to address this heterogeneity, we will conduct a series of sensitivity analyses restricted to subsets of studies that share the same classification scale and grouping, for instance (1) G0 vs. G≥1, (2) G0 vs. G≥2 (excluding G1), (3) G0–1 vs. G≥2, and (4) no hearing aids vs. use of hearing aids. This stepwise approach will allow us to assess how different phenotype definitions affect the genotype–phenotype association, while maintaining transparency, and may help promote clearer and more consistent case/control definitions in future genetic studies of PIHL. We acknowledge that these sub-analyses will reduce statistical power due to the smaller number of studies included in each.

We modified the re-analysis paragraph as follows: “For independent study populations in which the exact number of patients per ototoxicity grade is available, we will recategorize the data according to the different classification schemes used in the meta-analysis (e.g., G0 vs. G≥1, G0–1 vs. G≥2, G0 vs. G≥2 excluding G1) to maximise comparability.” (page 5, lines 267-269)

We modified the robustness assessment paragraph as follows: “Sensitivity analyses will be conducted whenever possible to assess the robustness of our findings. These will include stratifications by study quality, presence of non-genetic covariates (e.g., age at treatment, cranial radiation, platinum exposure, concomitant ototoxic drugs), and phenotype definition. The latter will consider how ototoxicity grades are handled (e.g., G0 vs. G≥1, G0–1 vs. G≥2, G0 vs. G≥2 excluding G1 and no hearing aids vs. use of hearing aids). If data allow, analyses stratified by ototoxicity grading system will be performed.” (page 6, lines 291-296)

We modified the discussion paragraph as follows: “A major limitation of this systematic review is the lack of consensus on validated paediatric ototoxicity scales, which assess either threshold changes from baseline or absolute hearing level. While baseline data are ideal, they are often missing, making absolute thresholds more commonly reported. Hearing data with baseline assessment will inform quality appraisal but not eligibility. To enable meta-analysis across studies using different grading systems, hearing loss will be dichotomized as present or absent, based on each study’s original classification. This inclusive strategy increases power but may introduce heterogeneity, particularly regarding the handling of Grade 1, which has variable clinical relevance. Although overall concordance between grading systems is

generally good [40], discrepancies persist, especially in early grades. To explore the impact of different phenotype definitions, a series of sensitivity analyses will be conducted as outlined in the methods. This approach aims to balance inclusivity with phenotypic clarity and may help inform future standards for case/control definitions in genetic studies of PIHL." (page 7, lines 346-357)

3. If the authors want to look at variants based on grade of hearing loss, they will need to do a dose and dose intensity exposure analysis (NOT cumulative dose exposure) since the amount of cisplatin exposure is important in concentration-dependent adverse reactions. Cumulative exposure is not relevant because hearing loss occurs and stabilizes before cumulative exposure is reached. The lack of serial audiograms for many European centres means they use cumulative exposure, but this is not a valid surrogate for when and the degree of hearing loss as previous papers have noted. It is recommended that the authors include this as a major limitation in their protocol (and most cisplatin-induced ototoxicity genetic association studies).

We thank the reviewer for this important clarification. We agree that dose intensity is more relevant than cumulative dose when investigating concentration-dependent toxicities like cisplatin-induced hearing loss, especially when analysing associations by severity grade. Hearing loss often occurs early during treatment and stabilizes, so cumulative dose may not accurately reflect the exposure that caused the damage.
Unfortunately, dose intensity is rarely reported in the studies we expect to include, often due to the lack of serial audiograms or detailed treatment schedules. When available, we will extract dose intensity data. Otherwise, cumulative dose will be extracted and used cautiously, with the understanding that it may not fully capture the timing or exposure relevant to phenotype severity.

We modified the discussion paragraph as follows: "Another important limitation of our study is the way cisplatin exposure is commonly reported. Although dose intensity is more relevant for assessing concentration-dependent toxicities such as PIHL [10], most studies report only cumulative dose. Therefore, when dose intensity data are unavailable, we will extract cumulative dose, while acknowledging that it may not accurately capture the timing or extent of exposure most relevant to hearing loss onset and severity. This limitation, which is shared by many genetic association studies on PIHL, should be carefully considered when interpreting our results." (page 7, lines 359-365)

Minor comments:

1. CTCAE has no "grade 0" group. Those who have no ototoxicity are classified as controls. Ototoxicity and other CTCAE adverse events start with Grade 1 and continue up from there. Please adjust this in the manuscript.

We thank the reviewer for this clarification. **We modified the terminology in Table 2** to reflect that individuals without ototoxicity are referred to as "controls" rather than "Grade 0," in alignment with CTCAE standards.
